# Impact of fasting on stress systems and depressive symptoms in patients with major depressive disorder: a cross-sectional study

**DOI:** 10.1038/s41598-022-11639-1

**Published:** 2022-05-10

**Authors:** Britta Stapel, Daniela Fraccarollo, Mechthild Westhoff-Bleck, Johann Bauersachs, Ralf Lichtinghagen, Kirsten Jahn, Alexandra Burkert, Vanessa Buchholz, Stefan Bleich, Helge Frieling, Xiao-Qi Ding, Kai G. Kahl

**Affiliations:** 1 Department of Psychiatry, Social Psychiatry and Psychotherapy, Hannover Medical School, Carl-Neuberg-Str. 1, 30625 Hannover, Germany; 2 Department of Cardiology and Angiology, Hannover Medical School, Hannover, Germany; 3 Institute for Clinical Chemistry, Hannover Medical School, Hannover, Germany; 4 Institute of Diagnostic and Interventional Neuroradiology, Hannover Medical School, Hannover, Germany

**Keywords:** Psychology, Metabolism

## Abstract

Major depressive disorder (MDD) is frequently associated with poor response to treatment. Common antidepressants target neurotransmission and neuronal plasticity, which require adequate energy supply. As imaging studies indicate disturbances in central energy metabolism, and caloric restriction improves neuroplasticity and impacts mood and cognition, correction of energy status might increase the effectiveness of antidepressant treatments and reduce the psychopathological symptoms of depression. Metabolic parameters, stress hormones, and brain-derived neurotrophic factor (BDNF) levels were assessed in serum of depressed inpatients (MDD, *N* = 21) and healthy volunteers (Ctrl, *N* = 28) before and after a 72 h fasting period during which only water was consumed. Depression severity was assessed by Beck’s Depression Inventory (BDI)-2 sum-score and cognitive-affective and somatic sub-scores. Fasting similarly impacted metabolic parameters and stress systems in both groups. Fasting elevated BDI-2 sum-scores and somatic sub-scores in Ctrl. In MDD, fasting increased somatic-, but decreased cognitive-affective symptoms. Sub-group analyses based on BDI-2 sum-scores pre-fasting showed that cognitive-affective symptoms decreased in patients with moderate/severe but not in those with mild symptoms. This was associated with differential changes in BDNF levels. In conclusion, fasting improved cognitive-affective sub-scores in MDD patients with moderate/severe symptoms that had not responded to prior therapy. Interventions that modulate energy metabolism might directly improve cognitive-affective symptoms and/or augment therapeutic efficacy in moderate-to-severely depressed patients.

## Introduction

Major depressive disorder (MDD) is a severe mental disease predicted by the World Health Organization to be the leading cause of disease burden by 2030^1^. Due to its multifactorial nature and heterogeneous symptomatology, determination of the precise etiology of MDD remains challenging.

An early hypothesis, based on the proposed cause of action of antidepressant drugs, suggested a depletion of monoamines in the central nervous system as the underlying pathomechanism of MDD^2^. However, despite the availability of a variety of different antidepressants that primarily target neurotransmission, these treatment options often fail to produce adequate results concerning response and remission^3–5^. In fact, up to 50% of all patients diagnosed with MDD display a therapy-resistant form of the disease^3,5^, indicating that alternative pathomechanisms, which are not directly targeted by classical antidepressant treatment, are likely to contribute to MDD development and progression, and that alternative treatment options are needed.

Imaging studies and subsequent meta-analyses proposed changes in neuronal plasticity to be critically involved in the pathology of MDD^6^ and decreased levels of neurotrophic factors, including brain-derived neurotrophic factor (BDNF), were previously reported^7^.

Importantly, maintenance of neuroplasticity requires an adequate energy supply and multiple studies have indicated that caloric restriction augments function and structure in the hippocampal region while high calorie intake appears to be detrimental (reviewed in^8^) and accordingly imaging studies found disturbances in brain energy metabolism in the context of MDD^9–11^.

In this regard, fasting is associated with a robust increase in blood ketone bodies, produced and secreted by hepatocytes during gluconeogenesis that rise in the circulation up to day three of fasting and plateau thereafter^12^. Ketones constitute an alternative cerebral energy source and in addition appear to impact brain function by induction of BDNF expression^13^. While depending on the protocol, caloric restriction was shown to impact cognitive function in rodent models, if applied in young animals, as indicated by increased learning and memory capacity, improved motor coordination and overall cognitive performance^14^. Additionally, fasting was found to be associated with alterations in mood, including worsened mood, heightened irritability, difficulties concentrating, and increased fatigue, as well as an increase in depressive score in mentally healthy humans^15–17^.

Given the interrelationship of neuronal plasticity and central as well as peripheral energy metabolism, one might speculate that antidepressant efficacy might be hampered by an adverse central energy situation and that in turn a correction of peripheral and subsequently cerebral energy status might be a prerequisite to enable the maximal efficacy of antidepressant therapies.

In the present study, the effect of a single 72 h fasting intervention on metabolic parameters and Beck’s Depression Inventory (BDI)-2 scores was compared in healthy volunteers (Ctrl) and MDD inpatients. As previous studies confirmed significant variations in MDD symptomology between patients, thereby highlighting the importance to address individual symptom patterns, we subsequently analyzed somatic and cognitive-affective sub-scores of BDI-2^18,19^. Importantly, prolonged fasting and resulting metabolic stress has previously been described to increase the activity of stress systems in non-depressed individuals^20–22^ and chronic activation of stress pathways are implicated in MDD etiology^23–27^. Therefore, circulating levels of norepinephrine (NE) as indicator for sympathetic activation, aldosterone as a parameter of the renin–angiotensin–aldosterone-system (RAAS) and cortisol as read-out for hypothalamic–pituitary–adrenal (HPA) axis activity were assessed in Ctrl and MDD groups at baseline and in response to fasting stress.

## Results

### Metabolic serum parameters are comparable in the Ctrl and MDD group pre- and post-fasting

Levels of glucose, insulin, triglycerides and ketones did not significantly differ in the Ctrl and MDD group at T1 (Table 1). Fasting significantly decreased glucose levels in both groups. However, glucose levels were higher in the MDD group at T2 when compared to Ctrl (Table 1). Fasting resulted in a significant reduction of insulin levels in the Ctrl group and tended to decrease insulin in the MDD group. Insulin levels did not significantly differ between Ctrl and MDD at T2. Triglyceride levels remained unchanged in response to fasting in Ctrl and MDD groups (Table 1). The detected increase in ketone levels in response to fasting was more pronounced in the Ctrl than in the MDD group where only a trend to higher values was observed. Consequently, ketone levels were significantly lower in the MDD group at T2 when compared to Ctrl (Table 1).

**Table 1:** Comparison of metabolic parameters pre- and post-fasting in Ctrl and MDD.

	Ctrl		MDD		Statistics		
	T1 (*N* = 28)	T2 (*N* = 28)	T1 (*N* = 21)	T2 (*N* = 21)	Interaction effect	Group effect	Fasting effect
Glucose (mmol/L)	5.0 ± 0.8	3.6 ± 0.5****	5.0 ± 0.4	4.0 ± 0.9^#^****	F(1, 47) = 3.57; *P* = .065	F(1, 47) = 2.51; *P* = .120	F(1, 47) = 90.50; *P* < .0001
Insulin (mU/L)	17.1 ± 18.5	5.3 ± 4.7****	11.4 ± 6.6	6.1 ± 3.2	F(1, 47) = 3.05; *P* = .087	F(1, 47) = .975; *P* = .329	F(1, 47) = 21.40; *P* < .0001
Triglycerides (mmol/L)	1.3 ± 1.0	1.2 ± 0.3	1.1 ± 0.6	1.3 ± 0.5	F(1, 47) = .879; *P* = .3533	F(1, 47) = .004; *P* = .950	F(1, 47) = .010; *P* = .920
Ketones (µmol/L)	80 ± 93	4,312 ± 1,860****	67 ± 78	857 ± 1,627^####^	F(1, 47) = 46.10; *P* < .0001	F(1, 47) = 45.72; *P* < .0001	F(1, 47) = 98.19; *P* < .0001

Table depicts mean values ± SD. RM two-way ANOVA was performed. Effect sizes for interaction and main effects are depicted. For post-hoc analyses, Sidak’s multiple comparison test was applied.

****P < .0001, versus corresponding T1; ^####^*P* < .0001, ^#^*P* < .05 versus corresponding Ctrl. *P* < .05 was considered to be statistically significant.

### Fasting elicits similar induction of stress systems in the Ctrl and MDD group

**Figure 1:**
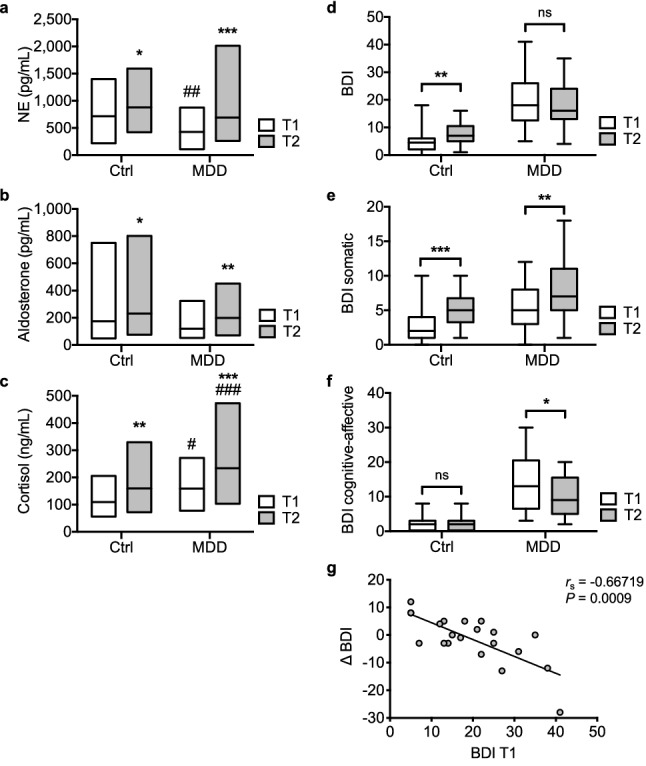
Effect of fasting on serum levels of stress hormones and BDI-2 scores in Ctrl and MDD. Box plots (**a**–**c**) depict mean with minimum and maximum values of norepinephrine (NE, **a**), aldosterone (**b**) and cortisol (**c**) before (T1) and after (T2) fasting in Ctrl and MDD. RM two-way ANOVA followed by Sidak’s multiple comparison test was used to compute adjusted *P*-values. Box plots (**d**–**f**) depict median and interquartile range and whiskers show minimum and maximum values of BDI-2 score (**d**), BDI-2 somatic sub-score (**e**) or BDI-2 cognitive-affective sub-score (**f**). *P*-values were computed using Wilcoxon matched-pairs signed rank test followed by Holm-Sidak test to correct for multiple comparisons. Only time-point differences were assessed (T1 versus T2) while potential differences between groups (Ctrl versus MDD) were not tested. Spearman correlation was used to assess association of BDI T1 and ΔBDI (**g**) in the MDD group. Resulting two-tailed *P*- and *r*_s_-values are depicted. ****P < .0001, ***P < .001, **P < .01, *P < .05 versus corresponding T1; ^####^*P* < .0001, ^###^*P* < .001, ^##^*P* < .01 versus corresponding Ctrl. Ctrl: *N* = 28, MDD: *N* = 21.

Measurement of circulating stress hormones revealed decreased levels of NE (Fig. 1a, Supplementary Table S1) as well as elevated cortisol levels (Fig. 1c, Supplementary Table S1) in the MDD group at T1. Contrarily, aldosterone levels were comparable in the Ctrl and MDD group at both time points (Fig. 1b, Supplementary Table S1). Fasting significantly increased all parameters in the Ctrl as well as in the MDD group. Cortisol levels remained elevated in the MDD group at T2 when compared to Ctrl (Fig. 1c, Supplementary Table S1), but no significant differences were observed in the respective delta values (difference in raw values for T2–T1), indicating comparable activation of analyzed stress parameters in the Ctrl and MDD group in response to 72 h metabolic fasting stress (Supplementary Fig. S1, Supplementary Table S2).

### Fasting distinctly impacts BDI-2 scores in the Ctrl and MDD group

Fasting stress resulted in a significant increase in BDI-2 score in the Ctrl but not in the MDD group (Fig. 1d, Supplementary Table S3). Fasting led to a similar increase in the somatic sub-score of BDI-2 in the Ctrl and the MDD group (Fig. 1e, Supplementary Table S3). Contrarily, fasting had no significant effect on the cognitive-affective sub-score of BDI-2 in the Ctrl group, indicating the observed increase in total BDI-2 score to be brought forward by the somatic sub-scale only. In the MDD group, fasting significantly lowered the cognitive-affective sub-score of BDI-2 (Fig. 1f, Supplementary Table S3).

### Fasting response in MDD patients depends on severity of symptoms at T1

The change in BDI-2 score (ΔBDI) in response to fasting showed a moderate to strong, negative association with the BDI-2 score at T1 (Fig. 1g). At T1 the median BDI-2 score in the MDD group was 18. Accordingly, two MDD sub-groups based on the BDI-2 score at T1, i.e. MDDlow (BDI ≤ 19, *N* = 11) and MDDhigh (BDI > 19, *N* = 10) were established, as a BDI-2 score > 19 indicates moderate to severe MDD, while a BDI-2 score ≤ 19 is indicative for mild MDD^28^. Both sub-groups did not differ significantly with regard to mean age, gender distribution, mean BMI, or antidepressant psycho-pharmacotherapy (Supplementary Table S4).

### Metabolic serum parameters are comparable in MDDlow and MDDhigh groups pre- and post-fasting

Glucose, insulin, triglycerides and ketone levels were similar in the MDDlow and MDDhigh group at T1 and T2 (Table 2). Glucose levels decreased significantly in both groups in response to fasting, while the observed decrease in insulin levels reached statistical significance only in the MDDlow but not in MDDhigh group. Triglyceride levels remained stable in response to fasting, and while a profound increase in ketone levels was observed in both groups, statistical significance was not reached (Table 2).

**Table 2:** Comparison of metabolic parameters in MDDlow in MDDhigh pre- and post-fasting.

	MDDlow		MDDhigh		Statistics		
	T1 (*N* = 11)	T2 (*N* = 11)	T1 (*N* = 10)	T2 (*N* = 10)	Interaction effect	Group effect	Fasting effect
Glucose (mmol/L)	4.9 ± 0.5	3.9 ± 0.9**	5.1 ± 0.3	4.1 ± 0.9*	F(1,19) = 0.018; *P* = 0.894	F(1,19) = 0.440; *P* = 0.515	F(1,19) = 20.09; *P* = 0.0003
Insulin (mU/L)	12.2 ± 7.7	6.4 ± 4.0*	10.6 ± 5.3	5.7 ± 2.3	F(1,19) = 0.099; *P* = 0.0756	F(1,19) = 0.420; *P* = 0.525	F(1,19) = 14.40; *P* = 0.0012
Triglycerides (mmol/L)	1.1 ± 0.4	1.2 ± 0.6	1.2 ± 0.7	1.3 ± 0.4	F(1,19) = 0.002; *P* = 0.964	F(1,19) = 0.409; *P* = 0.530	F(1,19) = 1.54; *P* = 0.229
Ketones (µmol/L)	55 ± 46	628 ± 1438	79 ± 104	1109 ± 1858	F(1,19) = 0.414; *P* = 0.528	F(1,19) = 0.473; *P* = 0.500	F(1,19) = 0.512; *P* < 0.036

Table depicts mean values ± SD. RM two-way ANOVA was performed. Effect sizes for interaction and main effects are depicted. For post-hoc analyses, Sidak’s multiple comparison test was applied.

**P < 0.01, *P < 0.05 versus corresponding T1. *P* < 0.05 was considered to be statistically significant.

### MDDlow and MDDhigh groups do not differ with regard to stress parameters at T1 and display a similar induction of stress systems in response to fasting

**Figure 2:**
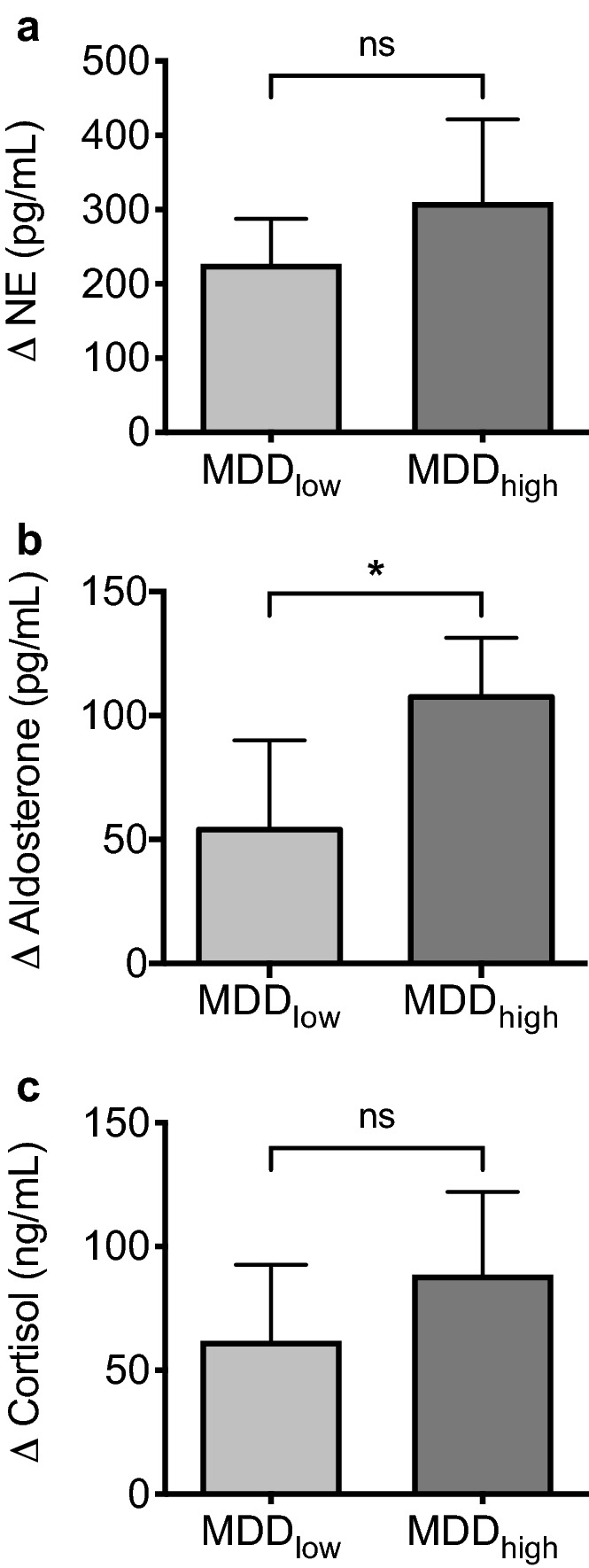
Fasting results in similar increases in stress parameters in MDDlow and MDDhigh. Bar graphs (mean with SEM) depict delta values showing the difference post- to pre-fasting (T2–T1) of norepinephrine (NE, **a**), aldosterone (**b**), and cortisol (**c**) in MDDlow (BDI ≤ 19 at T1) and MDDhigh (BDI > 19 at T1). Mann–Whitney test was used to calculate two-tailed *P*-values. *P < .05. MDDlow: *N* = 11, MDDhigh: *N* = 10.

At T1, analyzed stress parameters were comparable in the MDDlow and MDDhigh group. No significant differences were detected with regard to NE, aldosterone, or cortisol levels (Supplementary Table S5). Fasting resulted in a similar increase in NE and cortisol levels in both sub-groups. Contrarily, an augmented increase in aldosterone levels was observed in the MDDhigh when compared to the MDDlow group (Fig. 2a–c, Supplementary Table S6).

### Fasting improves cognitive-affective symptoms in the MDDhigh group

**Figure 3:**
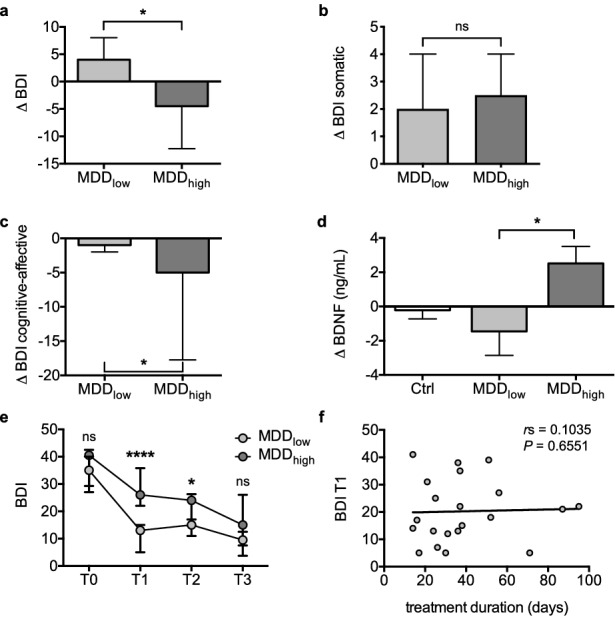
Effect of fasting on BDI-2 scores and BDNF serum levels depends on depression severity pre-fasting. Bar graphs (median with interquartile range) depict the delta of BDI-2 score (**a**), of BDI-2 somatic sub-score (**b**) or BDI-2 cognitive-affective sub-score (**c**) in MDDlow (BDI ≤ 19 at T1) or MDDhigh (BDI > 19 at T1) between post (T2) and pre (T1) fasting values. Change in BDNF values (ΔBDNF, mean with SEM) in Ctrl, MDDlow or MDDhigh are depicted in (**d**). Line chart shows median values and interquartile range of BDI-2 score over the time course of inpatient treatment from the start of inpatient treatment (T0) to the time of discharge (T3) in MDDlow (*N* = 10 for T3) and MDDhigh (*N* = 9 for T3; **e**). Spearman correlation was used to assess association of treatment duration before T1 with BDI-2 values at T1 in the MDD group (*N* = 21; **f**). Resulting two-tailed *P*- and *r*_s_-values are depicted. Two-tailed *P*-values comparing change in BDI-2 scores (**a**–**c**) as well as group differences of BDI-2 score (**e**) were assessed using Mann–Whitney test. One-way ANOVA followed by Tukey’s multiple comparison test was used in (**d**). ****P < .0001, *P < .05. Ctrl: *N* = 28, MDDlow: *N* = 11, MDDhigh: *N* = 10.

No significant change in BDI-2 score was observed in MDDlow or MDDhigh groups in response to fasting (Supplementary Fig. S2a, Supplementary Table S7). However, the change in BDI-2 score in response to fasting (ΔBDI) differed significantly between the MDDlow group that showed a slight increase, and the MDDhigh group that displayed decreased overall values (Fig. 3a, Supplementary Table S8), While the fasting-induced change in the somatic sub-score was comparable (Fig. 3b, Supplementary Table S8), the change in the cognitive-affective sub-score differed significantly between the MDDlow and MDDhigh group (Fig. 3c, Supplementary Table S8), with a significant decrease in the MDDhigh group only (Supplementary Fig. S2c, Supplementary Table S7).

### Fasting induces distinct changes in serum BDNF levels in MDDlow and MDDhigh groups

Measurements showed that the change in BDNF levels (ΔBDNF) brought forward by the fasting intervention was comparable in the Ctrl and the MDDlow group (Fig. 3d, Supplementary Table S8). However, ΔBDNF values significantly differed between the MDDlow group that displayed a trend to decreased values and the MDDhigh group, which showed an increased ΔBDNF value by comparison (Fig. 3d, Supplementary Table S8).

### Comparison of BDI-2 score, treatment response and treatment duration in the MDDlow and MDDhigh group

While MDDlow and MDDhigh groups did not differ in symptom severity at the start of inpatient treatment (T0), BDI-2 scores were significantly higher in the MDDhigh group at the start of the fasting intervention (T1, Fig. 3e, Supplementary Table S8). Consequently, the initial treatment response (% change in BDI-2 score) was significantly greater in MDDlow when compared to MDDhigh patients (Supplementary Table S4). Importantly, mean treatment duration leading up to the fasting intervention was comparable in MDDlow and MDDhigh groups (Supplementary Table S4) and did not correlate with BDI-2 values at T1 (Fig. 3f), indicating that differences in BDI-2 score at T1 could be attributed to different initial responses to guideline-based therapy. Additionally, overall treatment duration was comparable and treatment response from T1 to the time of discharge (T3) tended to be greater in the MDDhigh group when compared to the MDDlow group, without reaching statistical significance (Supplementary Table S4). Consequently, no significant difference was observed with regard to BDI-2 score at the time of discharge (Fig. 3e, Supplementary Table S8).

## Discussion

Our study revealed similar effects of a 72 h fasting intervention on peripheral parameters of metabolism and stress systems. Regarding psychometry, we found that fasting resulted in elevated somatic symptoms as well as overall BDI-2 scores in the healthy Ctrl group. In MDD patients, fasting was associated with a comparable increase in the somatic BDI-2 sub-score, while cognitive-affective symptoms decreased, resulting in overall comparable BDI-2 sum-scores pre- and post-fasting. Intriguingly, the beneficial effect of fasting on cognitive-affective symptomology could be attributed to a patient sub-group that started the fasting intervention with moderate to severe symptoms (BDI-2 score ≥ 19), which was associated with a differential regulation of circulating BDNF levels in this group.

Albeit the significant number of psycho-pharmacologic drugs available for MDD treatment, adequate, guideline-based therapy oftentimes fails to achieve response and remission, indicating underlying pathomechanisms that are not directly targeted by antidepressant treatment^3,5^. Imaging studies point to alterations in brain energy metabolisms in the context of depression^9–11,29^. Since neurotransmission as well as maintenance of neuroplasticity, the primary targets of antidepressant drugs, require adequate energy supply (reviewed in^8,30^), it appears reasonable that modulation of peripheral- and subsequently brain energy status might heighten the therapeutic efficacy of routinely used antidepressants.

### Effect of fasting on metabolic parameters

Fasting resulted in robust metabolic changes in both study groups that are in line with previous studies in mentally healthy individuals^12,31,32^. As described previously, fasting significantly increased circulating levels of ketones, which in turn have been shown to pass the blood–brain barrier thereby increasing metabolic efficacy by presenting an alternative energy substrate to glucose^12,17^. Additionally, robust evidence suggests a direct effect of ketones on the expression of neurotrophic factor BDNF and a subsequent beneficial impact on neuronal plasticity^13,33^.

### Effect of fasting on stress systems

Fasting induces metabolic stress that in turn was shown to trigger an activation of stress systems (HPA axis, RAAS, sympathetic activation)^20–22^, which were reported to be dysregulated in the context of MDD^23–27^. Therefore, fasting could impact MDD symptomology via these pathways. Plasma levels of the synaptic neurotransmitter NE constitute an indicator for sympathetic activation and central NE activity was found to be closely correlated to peripheral NE levels in healthy individuals as well as in MDD patients^34^. In contrast to previous studies, describing increased NE levels during an individual MDD episode^23–25^, we found significantly decreased NE levels in MDD patients pre-fasting. In our study, baseline values pre-fasting were obtained 6 ± 3 weeks after the beginning of inpatient therapy. Therefore, most MDD patients received pharmacotherapy (76%) that has previously been reported to impact NE levels in animal models^35^. Accordingly, MDD patients that received SSRIs displayed diminished NE levels at T1 when compared to patients that received SNRIs and/or NDRIs or were not treated with an antidepressant (data not shown), which might at least partly account for the observed overall decrease in the MDD group.

In line with studies by others that reported increased levels of cortisol in cerebrospinal fluid (CSF) and plasma of MDD patients, we found elevated cortisol levels in the MDD group pre- as well as post-fasting, indicating an increase in HPA axis activity^27^. Finally, aldosterone levels were comparable in the MDD and Ctrl group, which contrasts previous findings that mainly reported increased levels in the context of MDD^26,36,37^, although also decreased levels in suicide victims with MDD were described^38^. In line with previous studies in mentally healthy individuals^21,31^, fasting significantly increased all analyzed stress parameters and albeit cortisol levels remained elevated in the MDD group post-fasting, the induction was comparable in the Ctrl and MDD group, indicating a similar response to metabolic stress in the context of MDD.

### Effect of fasting on depression scores

In accordance to a previous study in healthy females^17^, fasting resulted in a significant increase in BDI-2 score in the Ctrl group, while no significant effect was observed in MDD patients. The opposite impact of fasting on somatic versus cognitive-affective sub-scores highlights the importance for a more detailed analysis of MDD symptoms^18^. The detected increase in BDI-2 somatic sub-score in the Ctrl as well as MDD group is in line with the observed comparable induction of stress systems. In accordance, the association of HPA axis activation and depressive symptoms has lately been attributed to somatic symptoms, while its association with cognitive-affective symptoms appeared weak^39^. Furthermore, the observed decrease in cognitive-affective symptoms in the MDD group is in line with potential positive effects of fasting brought forward by an increase in ketone levels and subsequent improvement of cerebral energy metabolism as well as by potential effects on BDNF expression^40,41^. The missing effect on the cognitive-affective sub-score in the Ctrl group might be explained by already low values at T1.

### Effect of fasting in MDD sub-groups based on symptom severity at the beginning of the fasting intervention

Analysis of MDD sub-groups based on BDI-2 score revealed differential effects in the MDDlow and MDDhigh group in response to fasting. The MDDlow group was defined by a greater response to guideline-based therapy compared to the MDDhigh group that responded poorly to initial treatment. In line with the observed similar effect of fasting on stress parameters, a comparable increase in somatic symptoms was observed in both MDD sub-groups^39^. Based on the observation that beneficial effects of fasting on cognitive-affective symptoms were limited to the MDDhigh group, the detected decrease in the cognitive-affective sub-score in MDD patients can be attributed to patients with more severe symptoms due to an insufficient response to prior guideline-based therapy. In line with the distinct change in cognitive-affective symptomology, significant differences in the change of BDNF levels in response to fasting, with a significantly greater delta value in MDDhigh patients, were observed^40^. While ketone levels that were shown to impact BDNF expression^13,33^, tended to be higher in the MDDhigh group following the fasting intervention, we did not find a direct correlation between ketone levels and BDNF values. BDNF levels were found to be decreased in unmedicated MDD patients when compared to healthy individuals^42,43^ and to be distinctly impacted by the response to antidepressant therapies, with a more prominent increase in values in individuals that responded to therapy than in non-responders^44,45^. As in our study fasting was initiated after most participants in the MDD group had started antidepressant treatment and as the MDDlow and MDDhigh groups are characterized by their distinct respective response to therapy, a potential ketone-mediated effect of fasting on BDNF levels might be masked in the MDDlow group that responded to therapy prior to fasting. Finally, as beneficial effects of fasting on the BDI-2 score appear to persist in the MDDhigh group after the fasting intervention, fasting might be a promising augmentation in the treatment of MDD patients that respond insufficiently to initial guideline-based therapy.

Of note, as a bidirectional relationship of MDD and medical conditions that are associated with abnormalities in peripheral metabolism and stress response such as cardiovascular and cardio-metabolic diseases are well established^46–53^, metabolic interventions might in parallel improve these common comorbidities. Finally, considering that neurotransmission as well as maintenance of neuroplasticity, which represent primary targets of antidepressant drugs, rely on adequate energy supply (reviewed in^8,30^), it appears reasonable that increasing ketone availability could represent a promising augmentative treatment option to directly improve cognitive-affective symptoms and to increase antidepressant efficacy.

## Conclusion

This pilot study highlights a beneficial effect of fasting primarily in MDD patients that suffered from more severe symptoms and did not sufficiently respond to initial antidepressant drug treatment. As the fasting intervention applied in this study resulted in an activation of stress pathways and a potentially related increase in somatic symptoms, alternate interventions that elicit an increase in ketone levels (i.e. ketogenic diet, exercise) without affecting stress systems might be discussed.

### Limitations

This study has several limitations. Due to ethical concerns, fasting in the MDD group was initiated 6 ± 3 weeks after the beginning of inpatient treatment. While this allowed for a detailed analysis of the fasting response in context of antidepressant efficacy and MDD symptom severity, it limited sample size in the corresponding sub-group analyses and resulted in a heterogeneous sample with regard to psychotropic drug treatment and severity of MDD symptoms at the beginning of the fasting period. Although the response to fasting on stress systems and peripheral metabolic parameters appeared comparable in the MDD and Ctrl group, an impact of psychotropic medication on these parameters at baseline or in response to fasting cannot be excluded. Additionally, sample size was limited in part due to the relatively high drop out (Supplementary Fig. S3) and psychometry was limited to the self-rated BDI-2 scale. Moreover, the MDD group was characterized by significantly higher BMI values and while BMI was comparable in MDDlow and MDDhigh sub-groups, it cannot be excluded that differences in BMI could have impacted results especially regarding peripheral metabolic- and stress parameters. Finally, our study focuses on stress parameters and neglects additional factors influenced by fasting that have been discussed to affect mood and cognition, namely the adipokines leptin and adiponectin^54,55^ as well as cytokines such as IL-6 and TNF-α^56–58^. Potential differences in the regulation of these factors in response to fasting between Ctrl and MDD as well as MDDlow and MDDhigh groups will be subject of further studies.

## Subjects and methods

### Subjects

The present study was approved by the local ethics committee at Hannover Medical School (ethics application number 6431) in accordance to the principles expressed in the Declaration of Helsinki, and all participants gave their written informed consent before entering the study. Our study follows a cross-sectional design and was mostly conducted in accordance to the respective The Strengthening the Reporting of Observational Studies in Epidemiology (STROBE) checklist^59^. Initially, 32 inpatients diagnosed with MDD using Diagnostic and Statistical Manual Fourth Edition (DSM-IV) criteria and 32 healthy volunteers (Ctrl) were included in the study from December 2013 till February 2017 to undergo a 72 h fasting intervention. Five patients of the MDD group and 4 individuals of the Ctrl group dropped out during the fasting intervention, data from 4 MDD patients were excluded from analyses due to a detected decrease of ketone levels post-fasting, indicating interruption of the fasting intervention, and two MDD patients were excluded from the analysis due to missing data. Therefore, data from 28 healthy control subjects and 21 MDD patients were included in the study, as depicted in the flowchart shown in Supplementary Fig. S3. All MDD patients were treated at the Department of Psychiatry, Social Psychiatry and Psychotherapy at Hannover Medical School and received cognitive-behavioral therapy. Additionally, 16 patients (76%) received antidepressant pharmaco-therapy (Supplementary Table S9). MDD diagnosis was confirmed by Structured Clinical Interview for DSM-IV for Axis I disorders (SCID-1). In the Ctrl group, MDD was excluded by SCID-1 or clinical interview. During the fasting intervention all participants completely stopped calorie intake and consumed only unflavored water for 72 h. Due to ethical concerns, fasting in the MDD group was initiated 6 ± 3 (mean ± SD) weeks after the start of inpatient treatment. The median BDI-2 score in the MDD group was 38 at the time of admission (T0), 18 at the beginning of fasting (T1) and 11 at the time of discharge (T3), and mean treatment duration was 9 ± 2 (mean ± SD) weeks. The following exclusion criteria applied for both groups: current or lifetime substance use disorder, schizophrenia, bipolar disorder, eating disorders, mental retardation, any acute physical disorder, in particular current or lifetime (auto-) immune disease, type-2 diabetes mellitus, cardiac diseases, systemic diseases, and current infectious disease. Ctrl and MDD group were comparable with regard to mean age and gender distribution while mean BMI was significantly higher in MDD when compared to Ctrl at T1 (Supplementary Table S9). Baseline BDI-2 score of the Ctrl and MDD group and effect of the fasting intervention on BDI-2 score as well as selected metabolic parameters of the Ctrl group were in part reported in prior studies that focused on magnetic resonance spectroscopic imaging of brain metabolism^17,29,60^.

### Clinical assessment and measurement of blood parameters

BDI-2 score was utilized to assess depression severity^28^. Based on previous works by others, cognitive-affective and somatic BDI-2 sub-scales were defined as follows: For the cognitive-affective sub-scale scores of items 1 to 14 (sadness, pessimism, past failure, loss of pleasure, guilty feelings, punishment feelings, self-dislike, self-criticalness, suicidal ideation, crying, agitation, loss of interest, indecisiveness, worthlessness) were summed, while items 15–21 (loss of energy, sleep problems, irritability, appetite problems, concentration, fatigue, loss of interest in sex) were added to calculated the somatic sub-scale^19^. BDI-2 scores in the MDD group were obtained at the time of admission (T0), pre-fasting (T1), post-fasting (T2) as well as at the time of discharge (T3). BDI-2 scores in the Ctrl group were assessed at T1 and T2.

For assessment of metabolic parameters and stress hormone levels, fasting blood was drawn at T1 and T2 between 07.00 and 08.00 am. Serum samples were stored on ice, followed by centrifugation within 60 min as indicated by the manufacturer. Serum aliquots were stored at -80 °C and subsequently used for detection of ketone bodies (ß-hydroxybutyrate, acetoacetate and acetone) by an enzymatic method (Wako Chemicals GmbH, Germany) in accordance to the manufacturer’s protocol. Concentrations of fasting glucose, insulin, and triglycerides were determined with established immunoassays (Roche Diagnostics, Germany). NE serum levels were measured by high-performance liquid chromatography with electrochemical detection. Commercially available ELISAs were used to determine serum levels of aldosterone and cortisol (Hölzel Diagnostika, Germany) and of BDNF (R&D Systems #DBD00, Germany) in accordance to the respective manufacturer’s instructions.

### Statistical analysis

*N*-numbers were calculated by power analysis for two-way, repeated measures (RM) ANOVA with within-between interaction using the G*Power 3.1 software^61^. A medium effect size was estimated based on three prior studies^17,29,60^. The following input parameters were used effect size f = 0.25, α error probability = 0.05, power (β error probability) = 0.8, number of groups = 2, number of measures = 2, correlation among RM = 0.5, and nonsphericity correction ε = 1. An overview regarding input and output parameters of the power analysis is depicted in Supplemental Table S10. All further statistical analyses were performed using GraphPad Prism 9.0 software. Shapiro–Wilk test was utilized to test for normal distribution. For comparison of two groups, if test for normal distribution was passed (alpha = 0.05), unpaired two-tailed t test was used. Unpaired t test with Welsh’s correction was performed when f test indicated significant differences in variances, and for non-normal distributed data Mann–Whitney test was utilized. Chi square test was used to compare gender distribution. For comparison of non-parametric BDI-2 data, *P*-values were computed using Wilcoxon matched-pairs signed rank test followed by Holm-Sidak test to correct for multiple comparisons. Only time-point differences were assessed. For comparison of more than two groups, one-way ANOVA followed by Tukey’s multiple comparison test or repeated measures (RM) two-way ANOVA followed by Sidak’s multiple comparison test were used as applicable. Correlation analyses were performed using Spearman’s rank-order correlation and Spearman’s correlation coefficient (*r*_s_) is reported. The interpretation of *r*_s_-values regarding the strength of the assessed associations follows recommendation of dedicated literature^62^.

Information regarding individual statistic tests is provided in the respective figure- and table legends and indicated supplemental tables. Differences were considered to be statistically significant for *P* < 0.05.

## Supplementary Information

Supplementary Information.
