# Supplementary material for: Impact of fasting on stress systems and depressive symptoms in patients with major depressive disorder: a cross-sectional study
Source: Sci Rep. 2022 May 10;12:7642. doi: 10.1038/s41598-022-11639-1 (PMC9091273; doi:10.1038/s41598-022-11639-1)
Supplement: Supplementary file 1 — Supplementary Information. [file 41598_2022_11639_MOESM1_ESM.pdf]

Impact of fasting on stress systems and depressive symptoms in patients with major depressive disorder – a cross-sectional study

Running title: Fasting in major depressive disorder

Britta Stapel [1], Daniela Fraccarollo [2], Mechthild Westhoff-Bleck [2], Johann Bauersachs [2], Ralf Lichtinghagen [3], Kirsten Jahn [1], Alexandra Burkert [1], Vanessa Buchholz [1], Stefan Bleich [1], Helge Frieling [1], Xiao-Qi Ding [4], Kai G. Kahl [1*]

[1] Department of Psychiatry, Social Psychiatry and Psychotherapy, Hannover Medical School, Hannover, Germany

[2] Department of Cardiology and Angiology, Hannover Medical School, Hannover, Germany

[3] Institute for Clinical Chemistry, Hannover Medical School, Hannover, Germany

[4] Institute of Diagnostic and Interventional Neuroradiology; Hannover Medical School, Hannover; Germany

Supplementary Results and Methods

Supplementary Results

The Supplementary Results section contains three supplementary tables and two supplementary figures regarding patient characteristics and additional data on the regulation of stress parameters and BDI-2 scores in Ctrl and MDD sub-groups in response to fasting. Additionally, six supplementary tables depict detailed results from statistical analysis corresponding to the figures in the main manuscript.

Supplementary Table 1: Effect sizes comparing stress hormone levels in CTRL and MDD pre- and post-fasting.

		**Statistics**		
	**Corresponding to**	**Interaction effect**	**Group effect**	**Fasting effect**
NE	Figure 1a	F(1,47)=1.50; *P*=.227	F(1, 47)=8.3;1 *P*=.006	F(1, 47)=25.78; *P*<.0001
Aldosterone	Figure 1b	F(1,47)=.493; *P*=.486	F(1, 47)=1.13; *P*=.293	F(1, 47)=17.53; *P*<.0001
Cortisol	Figure 1c	F(1,47)=.107; *P*=.0306	F(1, 47)=17.17; *P*=.0001	F(1, 47)=29.43; *P*<.0001

Results from RM two-way ANOVA are depicted. Effect sizes for interaction and main effects are shown. *P* < .05 was considered to be statistically significant.

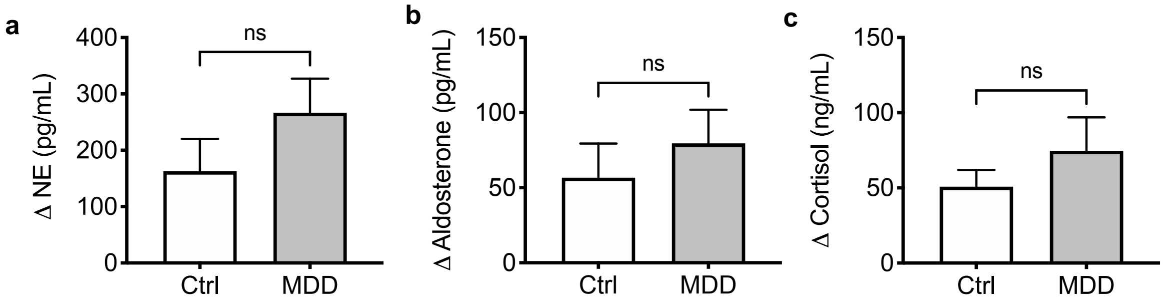

Supplementary Figure 1: Activation of stress pathways in Ctrl and MDD in response to fasting. Bar graphs (mean with SEM) depict increase in norepinephrine (NE, a), aldosterone (b) or cortisol (c) levels in Ctrl and MDD between post (T2) and pre (T1) fasting. Shapiro-Wilk test (alpha=.05) was used to test for normal distribution. Mann-Whitney test was utilized to compute two-tailed *P*-values. $P < .05$ was considered to be statistically significant. Ctrl: *N*=28, MDD: *N*=21.

Supplementary Table 2: Effect sizes comparing changes of stress hormone levels in CTRL and MDD in response to the fasting intervention

	**Corresponding to**	**Statistics**
ΔNE	Supplementary Figure 1a	U=261; P=.515
ΔAldosterone	Supplementary Figure 1b	U=253; P=.417
ΔCortisol	Supplementary Figure 1c	U=266; P=.582

Results from Mann-Whitney test are depicted. Two-tailed *P*-values were calculated. $P < .05$ was considered to be statistically significant.

Supplemental Table 3: Statistical analysis comparing BDI-2 scores in CTRL and MDD pre- and post-fasting.

		**Statistics**	
	**Corresponding to**	**Ctrl**	**MDD**
BDI sum score	Figure 1d	Z=-3.078; P=.001	Z=-.282; P=.790
BDI somatic	Figure 1e	Z=-3.629; P<.0001	Z=-3.056; P=.001
BDI cognitive-affective	Figure 1f	Z=-.659; P=.535	Z=-2.415; P=.014

Wilcoxon matched-pairs signed rank test was performed. Only time-point differences were assessed (T1 versus T2). Exact, two-tailed *P*-values before adjustment for multiple testing are depicted.

Supplementary Table 4: Characteristics of MDD_{low} and MDD_{high} groups.

	**MDD_{low} (*N*=11)**	**MDD_{high} (*N*=10)**	**Statistics**
Age (years)	37 ± 13 (19-53)	38 ± 14 (20-59)	$t(19)=.210$; $P=.836$ [a]
Gender	55% female	60% female	$\chi^2(1)=.0255$, $P=.874$ [b]
BMI (kg/m^2)	28 ± 5	29 ± 6	$t(19)=.239$; $P=.814$ [a]
Medication	SSRI: *N*=2 (18%)<br>SSRI + AGOM: *N*=2 (18%)<br>SNRI: *N*=0 (0%)<br>SNRI + NDRI: *N*=0 (0%)<br>SNRI + AGOM: *N*=1 (9%)<br>AGOM: *N*=1 (9%)<br>No drugs: *N*=5 (45%)	SSRI: *N*=3 (30%)<br>SSRI + AGOM: *N*=0 (0%)<br>SNRI: *N*=3 (30%)<br>SNRI + NDRI: *N*=1 (10%)<br>SNRI + AGOM: *N*=0 (0%)<br>AGOM: *N*=1 (10%)<br>No drugs: *N*=2 (20%)	
Treatment duration before fasting (T0-T1, weeks)	5 ± 2	7 ± 4	$t(19)=1.53$; $P=.142$ [a]
Duration of inpatient treatment (weeks)	9 ± 2 (*N*=10)	9 ± 3 (*N*=9)	$t(17)=.259$; $P=.799$ [a]
Change in BDI-2 from T0 to T1 (median, %)	-67	-34	$U=7.50$; $P=.0003$ [c]
Change in BDI-2 from T1 to T3 (median, %)	-29 (*N*=10)	-44 (*N*=9)	$U=35.0$; $P=.435$ [c]

Age, body mass index (BMI), and treatment duration are depicted as mean ± SD. Median values for percentage change of BDI-2 are shown. Shapiro-Wilk test was used to test for normal distribution. Unpaired, two-tailed t test (normal distribution, a) or non-parametric Mann-Whitney test was applied (c). Chi square test was used to compare gender distribution (b). Two-tailed *P*-values are depicted and $P < .05$ was considered to be statistically significant. AGOM: agomelatine, SNRI: serotonin-norepinephrine reuptake inhibitor, NDRI: norepinephrine and dopamine reuptake inhibitor; T0: start of inpatient treatment; T1: start of fasting intervention; T3: time of discharge.

Supplementary Table 5: Serum levels of stress hormones in MDD_{low} and MDD_{high} groups at T1.

	MDD_{low} (*N*=11)	MDD_{high} (*N*=10)	Statistics
NE (pg/mL)	426 ± 234	429 ± 185	*U*=55; *P*>.999 [b]
Aldosterone (pg/mL)	109 ± 61	130 ± 79	*U*=42; *P*=.387 [b]
Cortisol (ng/mL)	147 ± 47	173 ± 52	*t*(19)=1; *P*=.234 [a]

Means ± SD are depicted. Shapiro-Wilk test was used to test for normal distribution. Unpaired, two-tailed t test (normal distribution, a) or Mann-Whitney test (b) were used to compute two-tailed *P*-values. $P < .05$ was considered to be statistically significant. NE: norepinephrine.

Supplementary Table 6: Effect sizes regarding changes of stress hormone levels in MDD_{low} and MDD_{high} in response to fasting.

	Corresponding to	Statistics
ΔNE	Figure 2a	*U*=51; *P*=.809
ΔAldosterone	Figure 2b	*U*=22; *P*=.020
ΔCortisol	Figure 2c	*U*=39; *P*=.282

Results from Mann-Whitney test are depicted. Two-tailed *P*-values were calculated. $P < .05$ was considered to be statistically significant.

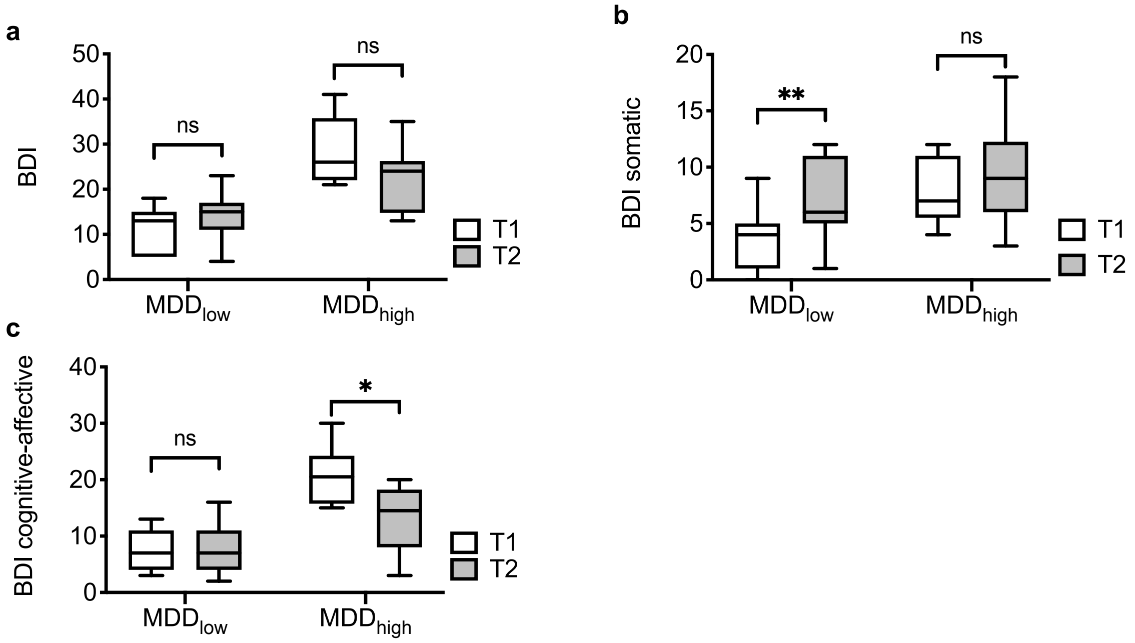

Supplementary Figure 2: Effect of fasting on BDI-2 scores in MDD$_{low}$ and MDD$_{high}$ patients. Box plots depict median and interquartile range and whiskers show minimum and maximum values of BDI-2 sum score (a), BDI-2 somatic sub-score (b) or BDI-2 affective-cognitive sub-score (c) in MDD$_{low}$ and MDD$_{high}$. *P*-values were computed using Wilcoxon matched-pairs signed rank test followed by Holm-Sidak test to correct for multiple comparisons. Only time-point differences were assessed (T1 versus T2). **P <.001; $^{*}P < .05$ versus corresponding T1. MDD$_{low}$: *N*=11, MDD$_{high}$: *N*=10.

Supplementary Table 7: Statistical analysis comparing BDI-2 scores in MDD$_{low}$ and MDD$_{high}$ pre- and post-fasting.

		**Statistics**	
	**Corresponding to**	**MDD$_{low}$ (*N*=11)**	**MDD$_{high}$ (*N*=10)**
BDI sum score	Supplementary Figure 2a	*Z*=-1.791; *P*=.076	*Z*=-1.836; *P*=.074
BDI somatic	Supplementary Figure 2b	*Z*=-2.732; *P*=.005	*Z*=-1.546; *P*=.137
BDI cognitive-affective	Supplementary Figure 2c	*Z*=-.410; *P*=.734	*Z*=-2.502; *P*=.010

Wilcoxon matched-pairs signed rank test was performed. Only time-point differences were assessed (T1 versus T2). Exact, two-tailed *P*-values before adjustment for multiple testing are depicted.

Supplemental Table 8: Effect sizes regarding changes in BDI-2 sum- and sub-scores and BDNF levels in MDD_{low} and MDD_{high} in response to fasting.

	**Corresponding to**	**Statistics**
ΔBDI sum score	Figure 3a	*U*=23; *P*=.022 [a]
ΔBDI somatic	Figure 3b	*U*=51.5; *P*=.822 [a]
ΔBDI cognitive-affective	Figure 3c	*U*=20; *P*=.012 [a]
ΔBDNF	Figure 3d	F(2, 46)=4.033; *P*=.024 [b]
BDI time course	Figure 3e	T0: *U*=37; *P*=.214 [a]<br>T1: *U*=0; *P*<.0001 [a]<br>T2: *U*=20; *P*=.012 [a]<br>T3: *U*=24.5; *P*=.098 [a]

Results from Mann-Whitney test (a) or one-way ANOVA (b) are depicted. Two-tailed *P*-values were calculated. $P < .05$ was considered to be statistically significant.

Supplementary Table 9: Characteristics of Ctrl and MDD groups.

	**Ctrl (*N*=28)**	**MDD (*N*=21)**	**Statistics**
Age (years)	42 ± 10 (22-66)	38 ± 14 (19-59)	$t(47)=1.37$; $P=.177$ [a]
Gender	68% female	57% female	($\chi^2(1)=.593$; $P=.414$ [b]
BMI (kg/m^2)	25 ± 4	28 ± 6	$t(31.7)=2.29$; $P=.029$ [c]
Medication	No drugs: *N*=28 (100%)	SSRI: *N*=5 (24%)<br>SSRI + AGOM: *N*=2 (9.5%)<br>SNRI: *N*=3 (14%)<br>SNRI + NDRI: *N*=1 (5%)<br>SNRI + AGOM: *N*=1 (5%)<br>NDRI: *N*=0 (0%)<br>AGOM: *N*=2 (9.5%)<br>No drugs: *N*=7 (33%)	

Values for age and body mass index (BMI) are depicted as mean ± SD. Shapiro-Wilk test was used to test for normal distribution. If test for normal distribution was passed (alpha = .05), unpaired two-tailed t test was used (a). Unpaired t test with Welsh's correction was performed, when f test indicated significant differences in variances (c). Chi square test was used to compare distribution of gender between groups (b). Two-tailed *P*-values are depicted and $P < .05$ was considered statistically significant. AGOM: agomelatine, SNRI: serotonin-norepinephrine reuptake inhibitor, NDRI: norepinephrine and dopamine reuptake inhibitor.

Supplementary Methods

The Supplementary Method section contains an additional figure providing an overview regarding lost samples in the Ctrl and MDD group as well as a table detailing parameters of the underlying power analysis performed for this study.

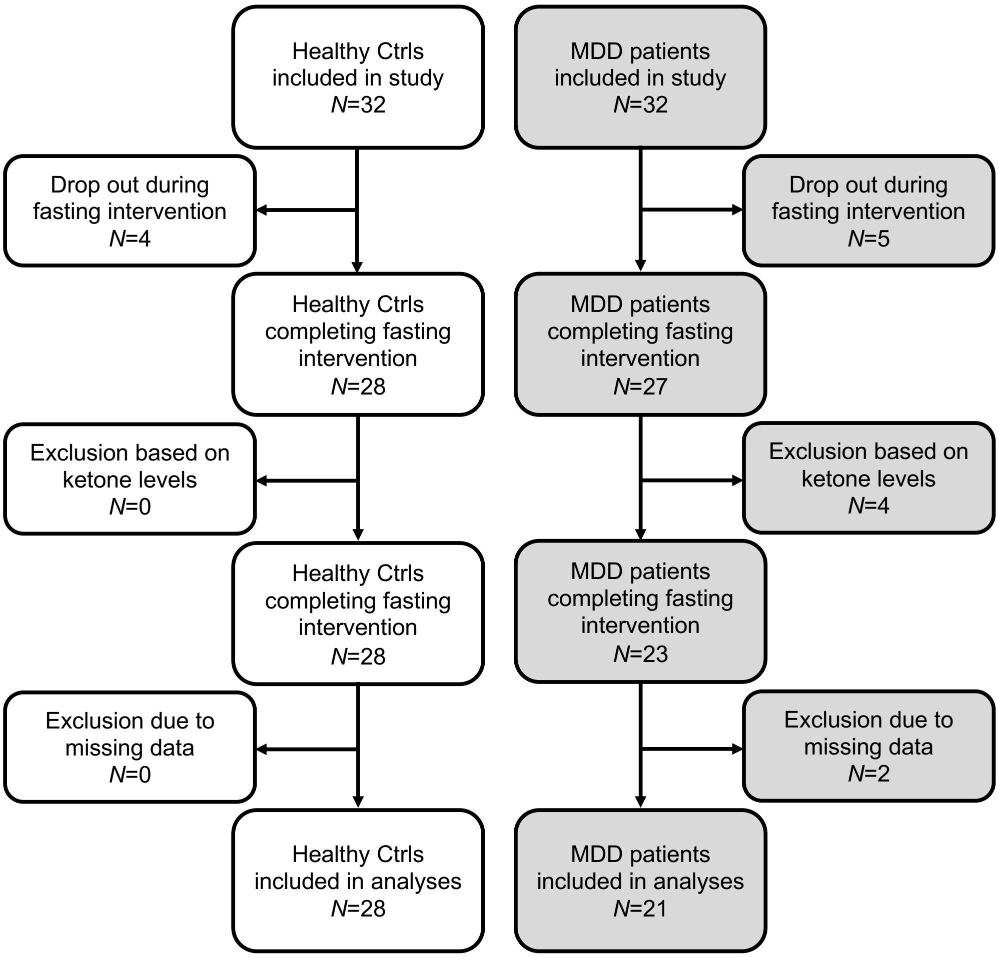

Supplementary Figure 3: Flowchart detailing lost samples in Ctrl and MDD groups.

Supplementary Table 10: Parameters of power analysis

Test family	F test		
Statistical test	ANOVA: RM, within-between interaction		
Input parameters:		Output parameters:	
Effect size f	0.25	Noncentrality parameter λ	8.50
α error probability	0.05	Critical F	4.15
Power (β error probability)	0.8	Numerator df	1
Number of groups	2	Denominator df	32
Number of measures	2	Total sample size	34
Correlation among RM	0.5	Actual power	0.81
Nonsphericity correction ε	1		

Power analysis performed with G*power software [(1)]. RM, repeated measures.

Supplementary References

1. Faul F, Erdfelder E, Lang AG, Buchner A. G*Power 3: a flexible statistical power analysis program for the social, behavioral, and biomedical sciences. *Behav Res Methods* 2007; **39**(2)**:** 175-191.
